# Nociception, Transcriptomics ET CETERA: NOCICEPTRA

**DOI:** 10.1007/s00424-022-02673-z

**Published:** 2022-02-24

**Authors:** Natja Haag, Angelika Lampert

**Affiliations:** 1 Institute of Human Genetics, Uniklinik RWTH Aachen University, Pauwelsstr. 30, 52074 Aachen, Germany; 2 Institute of Physiology, Uniklinik RWTH Aachen University, Pauwelsstr. 30, 52074 Aachen, Germany

Induced pluripotent stem cells (iPSCs) are an established cellular system to study healthy and diseased conditions in a human context in vitro. Sensory neurons and signaling mechanisms mediating the perception of pain share many similarities but also substantial differences between mice and humans, and thus, translating the findings from rodents to humans is not always straightforward [7]. Although human iPSC-derived nociceptors cannot fully recapitulate the development of the human nociceptive pathway, the study by Zeidler et al. [10], recently published in *Advanced Science*, tries to fill this gap. In their comprehensive, data-filled paper, the group of Michaela Kress (Institute of Physiology, Medical University Innsbruck) investigated iPSC-derived human nociceptor (iDN) development in the dish. Their study provides novel insights into and new data for (i) global gene expression during stem cell differentiation into sensory neurons, (ii) novel miRNA candidates regulating distinct stages of iDN differentiation, (iii) disease onset or susceptibility windows for disease-associated gene sets, and (iv) bundles all this information in the searchable online tool NOCICEPTRA.

Using a small molecule–based differentiation method modified from Chambers et al. [1], they first followed neuron induction and maturation over a time frame of 36 days (D36), sampling RNA at six different time points for analysis by a combined long and short RNA sequencing approach to cover long protein-coding mRNA as well as short microRNA transcripts. Their initial protein-coding mRNA analysis shows successful nociceptor differentiation and identified 6 hub-gene networks involving genes relevant for multifunctional neuronal and synaptic development and neural tube or neural crest genes. As expected, pluripotency markers were strongly downregulated during differentiation, whereas neural crest cell markers peaked around D10 to D15, and many nociceptive markers were continuously upregulated. The transcriptome of differentiated neurons was more similar to that of dissociated human dorsal root ganglion (DRG) cultures than of whole ganglia, which is most likely due to the fact that DRGs comprise of much more than only sensory neurons, e.g., satellite glial cells.

Using trajectory analysis, they were able to identify five different iDN differentiation stages: A pluripotency stage which similarly to the early differentiation stage (followed by the neural progenitor stages) continuously decreases during differentiation by measures of marker gene expression, while the nociceptor genes are upregulated during nociceptor and maturation stages. Interestingly, there are two complementary groups which seemingly inverse gene regulation: early/late maturation genes and those in the early neural progenitor group. Thus, the presented data convincingly recapitulate the stages of nociceptor differentiation as described for mice [2, 3, 6] suggesting that nociceptor fate determination is common among rodents and humans. Nociceptor subtype specification and diversification is also represented by the dataset as shown, e.g., by an increase of nociceptor-specific ion channel expression from D16-D36. Here a single cell–based analysis would add more detail and clarity regarding the sensory neuron populations that evolve during human development.

Several recent studies performed single cell transcriptomic analysis on DRGs of non-human primates (macaque) [4] and humans [9] and revealed significant sex differences and inter-species divergence among specific nociceptor subsets in human DRG subpopulation transcriptomes. For example, whereas mice display an enriched expression of SCN9a in nociceptors, macaque SCN9A is broadly expressed at similar levels in all neuronal types. Zeidler et al. did not directly address these aspects, although the iPSC lines they used derived from two females and one male donor, and comparisons may thus have been fruitful. Differentiation of sensory neurons using the Chambers protocol shows high variability [5, 8] and thus the choice of clones may also affect the outcome, which is an important factor to consider when discussing RNAseq results from differentiated cells.

Beyond mRNA transcriptomic analysis, Zeidler et al. set out to also identify the expression trajectories of small non-coding microRNAs (miRNAs) whose many regulatory functions we only start to understand. Zeidler and colleagues establish a link between their findings from coding and non-coding RNA analysis: they identified hub genes that are most likely important (if not the most important) regulators of the modules, and the authors additionally performed a miRNA:mRNA target analysis for those hub genes identified. Thereby they identified miRNAs which e.g. regulate pre- and post-synapse development via hub genes, which can potentially also act as master switches, and miRNAs with impact on neurite outgrowth modulation. This interesting approach could in the future also be expanded to long non-coding RNAs (lncRNA), as they are of increasing importance for nociceptor differentiation. The author’s data set already covers lncRNAs and could easily be implemented within their online tool NOCICEPTRA as a searchable RNA.

To make the complex data and their analysis pipelines accessible and manageable for other researchers, the authors developed the mentioned online tool NOCICEPTRA to analyze and visualize time trajectories of genes and miRNAs of interest. This valuable tool allows for general exploration of gene and miRNA expression, hub modules and pathways as well as disease susceptibility windows with the option to specifically zoom into genes of interest, their expression trajectory during nociceptor differentiation and potential regulation by miRNAs. With the increasing availability of big data, such tools become more and more important and will hopefully in the future also include state-of-the art single cell and single nuclear sequencing data and approaches.
